# Hourly fetal urine production rate in isolated oligohydramnios at term

**DOI:** 10.1371/journal.pone.0250659

**Published:** 2021-05-21

**Authors:** Hyun-Joo Seol, Ho Yeon Kim, Geum-Joon Cho, Min-Jeong Oh

**Affiliations:** 1 Department of Obstetrics & Gynecology, College of Medicine, Kyung Hee University, Seoul, Korea; 2 Department of Obstetrics & Gynecology, College of Medicine, Korea University, Seoul, Korea; Kobe University Graduate School of Medicine School of Medicine, JAPAN

## Abstract

**Objective:**

The aim of this study was to evaluate the hourly fetal urine production rate (HFUPR) via three-dimensional ultrasonography in women with isolated oligohydramnios and compare with normal pregnant women at term.

**Materials and methods:**

This was a prospective observational cohort study of 112 women from 34 to 40 6/7 weeks’ gestation. They were classified into three groups according to the amniotic fluid index (AFI) and ultrasonographic estimated fetal weight (EFW) as isolated oligohydramnios (defined as AFI below 5% and appropriate EFW corresponding to gestational age) (n = 34) and IUGR (defined as EFW below 5% corresponding to gestational age irrespective amniotic fluid) (n = 17), and normal pregnancy (n = 61). HFUPR was measured using three-dimensional virtual organ computer-aided analysis. Adverse perinatal outcomes in all participants were examined.

**Results:**

There was no significant difference in HFUPR between patients with isolated oligohydramnios and women with normal pregnancies (median, 40.0 mL/h [interquartile range [IQR] 31.0–66.5] vs. 48.6 [31.5–81.2], p = 0.224). HFUPR was significantly decreased in the IUGR group (13.8 mL/h [IQR 10.1–24.8]), compared to the normal pregnancy group (p<0.001) and the isolated oligohydramnios group (p<0.001). HFUPR was significantly decreased in neonates with adverse perinatal outcomes compared to the control (24.7 mL/h [IQR 13.4–47.4] vs. 43.6 [29.8–79.0], p = 0.016).

**Conclusion:**

HFUPR was not decreased in patients with isolated oligohydramnios but was decreased in patients with IUGR when compared to normal controls at term.

## Introduction

Oligohydramnios is associated with uteroplacental insufficiency and fetal compromise and has been a component of fetal wellbeing tests for three decades [1]. It is associated with perinatal morbidity and mortality [2, 3] and cesarean delivery due to fetal distress [4]. Adverse perinatal outcomes were generally reported if oligohydramnios was combined with intrauterine growth restriction (IUGR) or if it developed in an earlier gestation [5–7]. It is usually called isolated oligohydramnios when only the amniotic fluid was decreased at term without any other fetal or maternal abnormality such as IUGR, fetal anomaly, and maternal hypertensive disease. Intensive monitoring and delivery have been warranted in isolated oligohydramnios to reduce perinatal mortality, but the association between isolated oligohydramnios and the adverse perinatal outcome is controversial [8–11]. It has been reported that perinatal morbidity of isolated oligohydramnios might be iatrogenic due to labor induction than oligohydramnios itself [12].

Fetal urine is a major source of amniotic fluid in the third trimester of pregnancy. Thus oligohydramnios in uteroplacental insufficiency has been thought to be associated with decreased fetal urine production in response to chronic fetal hypoxia, although the underlying mechanism is not fully understood [13, 14]. However, in isolated oligohydramnios it still remains uncertain how amnionic fluid is decreased. Since amniotic fluid volume is dynamically regulated by fetal urine, lung fluid, fetal swallowing, and intramembranous absorption, decreased amniotic fluid does not always indicate decreased fetal urine production [15, 16]. According to an experimental study, variation in intramembranous absorption may contribute to amnionic fluid abnormality [17]. As in the case of isolated oligohydramnios, one of its pathophysiology has been suggested to increased absorption of amniotic fluid due to alterations in the expression of aquaporins in amnion and placenta rather than decreased fetal renal blood flow [18, 19]. However, few reports have described about the fetal urine production in isolated oligohydramnios at term. Nowadays, the fetal urine production rate could be estimated by determining bladder volume using more sophisticated three-dimensional (3D) ultrasonography [20, 21]. The virtual organ computer-aided analysis (VOCAL) is a volumetric calculation technique using 3D which analyzing volume by rotating the axis of an object. The VOCAL method shows similar accuracy to the multiplanar mode in evaluating irregularly shaped objects but is faster and more convenient in clinical practice [22].

The aim of this study was to determine whether the hourly fetal urine production rate (HFUPR) on 3D ultrasonography is decreased in term fetuses with isolated oligohydramnios compared to those of normal pregnancy. We also aimed to examine whether a decrease in fetal urine production is observed in uteroplacental insufficiency by comparing the HFUPR in fetuses with IUGR and those in fetuses with normal pregnancy. Additionally, we analyzed any association between HFUPR and perinatal outcomes assuming that a decrease in HFUPR reflects intrauterine fetal hypoxia.

## Materials and methods

This prospective observational cohort study was enrolled between November 2011 and September 2013 at Korea University Guro Hospital in Seoul, Korea. This study was reviewed and approved by the Institutional Review Board of Korea University Guro Hospital (approval No. KUGH10185). Written informed consent was submitted by all participants when they were enrolled. All singleton pregnant women between 34 weeks’ and 40 6/7 weeks’ gestation who had regular antenatal care during the study period were included. Exclusion criteria were suspected fetal anomalies and maternal conditions which could affect amniotic fluid, such as preeclampsia, hypertension, diabetes, renal disease, lupus and other maternal diseases. Oligohydramnios caused by premature rupture of membranes was also excluded. All enrolled women reviewed symptoms of ruptured membranes; when membrane rupture was suspected, a sterile speculum examination was performed and the nitrazine test was performed. If gross leakage of fluid was observed or the pH was above 7.0, rupture of membrane was diagnosed.

Ultrasonographic examinations were performed with a Voluson E8 (General Electric healthcare, Austria) scanner using a 4-7-MHz curved array transducer. Oligohydramnios was diagnosed if the amniotic fluid index (AFI) was below the fifth percentile for the corresponding gestational age [23], and IUGR was diagnosed if the estimated fetal weight (EFW) was below the fifth percentile for gestational age. All measurements of AFI and EFW were performed in triplicate and averaged. All participants were classified into the following three groups: 1) a normal group comprised of those with a normal AFI and EFW; 2) an isolated oligohydramnios group, comprised of those with oligohydramnios but normal EFW; and 3) an IUGR group, comprised of those with an EFW below the fifth percentile for gestational age irrespective of amniotic fluid.

HFUPR was calculated as follows. Bladder volume was measured by 3D ultrasonography. The rotational VOCAL method, which creates slices of the bladder (each 30°) around the main axis of the bladder outline along with bladder contours manually traced on each of these six slices, were used to measure the fetal bladder volume. Then, the computer program automatically constructed a 3D model of the bladder and calculated the volume of this 3D model. At least two increasing bladder volume acquisitions were recorded at intervals of 5–10 min. If the second bladder volume was smaller than the first volume, then a third bladder volume was measured and the second bladder volume was regarded as the first bladder volume. Bladder volumes were measured repetitively until a larger volume was obtained. HFUPR was calculated by the following equation;

$$\begin{array}{l} \text{HFUPR}\;(\text{mL/h})=(\text{second}\;\text{bladder}\;\text{volume}\;\text{-}\;\text{first}\;\text{bladder}\;\text{volume)}\times (\text{60/x})\\ \left(\text{x}\;\text{is}\;\text{the}\;\text{time}\;\text{interval}\;\text{in}\;\text{minutes}\;\text{between}\;\text{bladder}\;\text{volume}\;\text{measurements}.\right) \end{array}$$

HFUPR was measured twice and the larger HFUPR was used for analysis. A single examiner performed all measurements to eliminate interobserver variation. The examiner was an expert with more than 7 years of experience in obstetrics and gynecology ultrasound, and with an extensive experience in volume measurement using VOCAL in pelvic pathology. To analyze intraobserver variation, three measurements of each HFUPR were performed in 37/112 women. Maternal age, parity, gestational age at diagnosis and delivery, and birth weights were recorded. Labor induction was performed according to the clinician’s preference. Adverse perinatal outcome was defined as the occurrence of any of the following events: cesarean section due to fetal distress, admission to neonatal intensive care unit (NICU), Apgar score below 7 at 5 mins and perinatal death. Previous report by Lee et al. has suggested that median HFUPR in normal pregnancy was 57.2 mL/h between 36 and 37 6/7 weeks of gestation in Korean women [20]. It was assumed that HFUPR might be decreased to lower quartile in isolated oligohydramnios or IUGR. Under this assumption, calculated sample size was 60 in normal group and 30 in isolated oligohydramnios or IUGR, respectively (assuming α = 0.05, power = 0.80 and 2:1 group sizes).

All statistical analyses were performed using SPSS 24.0 (SPSS Inc., Chicago, IL, USA) and R software packages (R version 3.5.3). Clinical variables were expressed as the mean ± SD or percentage when appropriate. HFUPR were expressed as the median with interquartile range (IQR). For analysis of differences between two groups, the Student’s t-test, Chi square test, Mann-Whitney U test or Fisher’s exact test were used as appropriate. The comparison of HFUPR in the three groups was analyzed by the Kruskal-Wallis test. The Bonferroni correction was used as post-hoc analysis. To determine intraobserver variability, the intraclass correlation coefficient was calculated. P values less than 0.05 were considered significant.

## Results

A total of 112 singleton fetuses were investigated. There were 61 women in the normal group, 34 in the isolated oligohydramnios group, and 17 in the IUGR group. Table 1 summarizes the demographic characteristics of each group. Maternal age did not differ between the normal, isolated oligohydramnios and IUGR groups. Nulliparous women comprised almost half the normal group but were significantly more common in other groups. The three groups did not differ with regard to gestational age at ultrasonographic measurement or delivery. Neonatal birth weight and AFI differed significantly among the groups. The IUGR group had lower birth weights than the normal pregnancy group (p<0.001) and the isolated oligohydramnios group (p<0.001). All neonatal birth weights were below the fifth percentile according to gestational age in the IUGR group. The IUGR group had the highest induction rate, followed by the isolated oligohydramnios group and the normal group.

**Table 1:** Demographic characteristics of the study group.

	Normal (n = 61)	Isolated oligohydramnios (n = 34)	IUGR (n = 17)	p
**Age (years)**	32.0±4.3	32.1±3.0	30.4±3.7	0.140
**Nulliparous, % (n)**	47.5 (29)	76.4 (26)^a^	94.1 (16)^a^^b^	0.001
**GA at measurement (weeks)**	37.9±0.9	37.7±2.2	37.7±1.2	0.699
**GA at delivery (weeks)**	39.0±1.2	38.9±1.8	38.5±1.3	0.354
**Birth weight (g)**	3280±380	3060±390^a^	2250±320^a^^b^	<0.001
**AFI (mm)**	129±30	55±17^a^	78±35^a^	<0.001
**Induction rate, % (n)**	14.8 (9)	42.9 (15)^a^	88.2 (15)^a^^b^	<0.001

Data are shown by mean±standard deviation.

^a^p-value <0.05 compared with the normal group.

^b^p-value <0.05 compared with the isolated oligohydramnios group.

GA, gestational age; AFI, amniotic fluid index.

**Fig 1:**
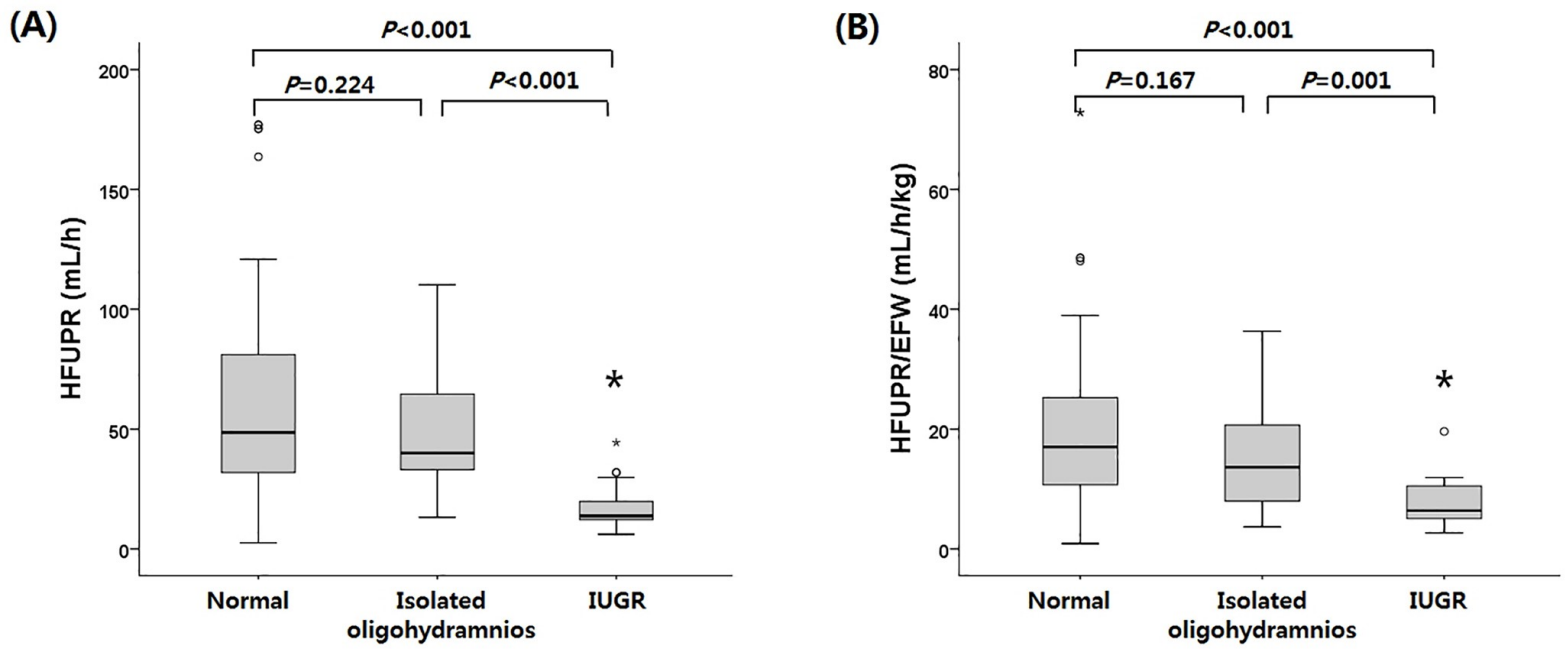
Hourly fetal urine production rate (HFUPR) according to amniotic fluid level and fetal weight. *Compared with the normal group and isolated oligohydramnios group. (A) HFUPR in each group. (B) HFUPR/EFW in each group (EFW, estimated fetal weight).

There was no significant difference in HFUPR between the normal and isolated oligohydramnios groups (median, 48.6 mL/h [IQR 31.5–81.2] vs. 40.0 [31.0–66.5], p = 0.224) (Fig 1). HFUPR were significantly decreased in the IUGR group (median, 13.8 mL/h [IQR 10.1–24.8]) compared to the normal and the isolated oligohydramnios groups (p<0.001; p<0.001, respectively). Because estimated fetal body weights were significantly different between the normal weight groups and the IUGR group, we calculated HFUPR per estimated fetal weight (HFUPR/EFW). There was no significant difference in HFUPR/EFW between the normal and isolated oligohydramnios groups (median, 17.0 mL/h/kg [IQR 10.7–25.4] vs. 13.7 [7.9–21.3], p = 0.167). HFUPR/EFW was significantly decreased in the IUGR group (median, 6.4 mL/h/kg [IQR 4.8–10.9]) compared to the normal group and the isolated oligohydramnios group (p<0.001; p = 0.001, respectively).

Table 2 shows the rate of adverse perinatal outcomes for each group. The adverse perinatal outcomes were occurred in 6% of normal group, 35.3% of isolated oligohydramnios group and 64.7% of IUGR group. There was no perinatal death or Apgar score below 7 at 5 mins.

**Table 2:** Adverse perinatal outcomes of the study group.

	Normal (n = 61)	Isolated oligohydramnios (n = 34)	IUGR (n = 17)	p
**Adverse perinatal outcomes, % (n)**	9.8 (6)	35.3 (12)	64.7 (11)	<0.001
**C/S due to fetal distress, n**	1	0	2	
**NICU admission, n**	5	12	11	
**Apgar score < 7 at 5 mins, n**	0	0	0	
**Perinatal death, n**	0	0	0	

C/S, cesarean section; NICU, neonatal intensive care unit.

**Fig 2:**
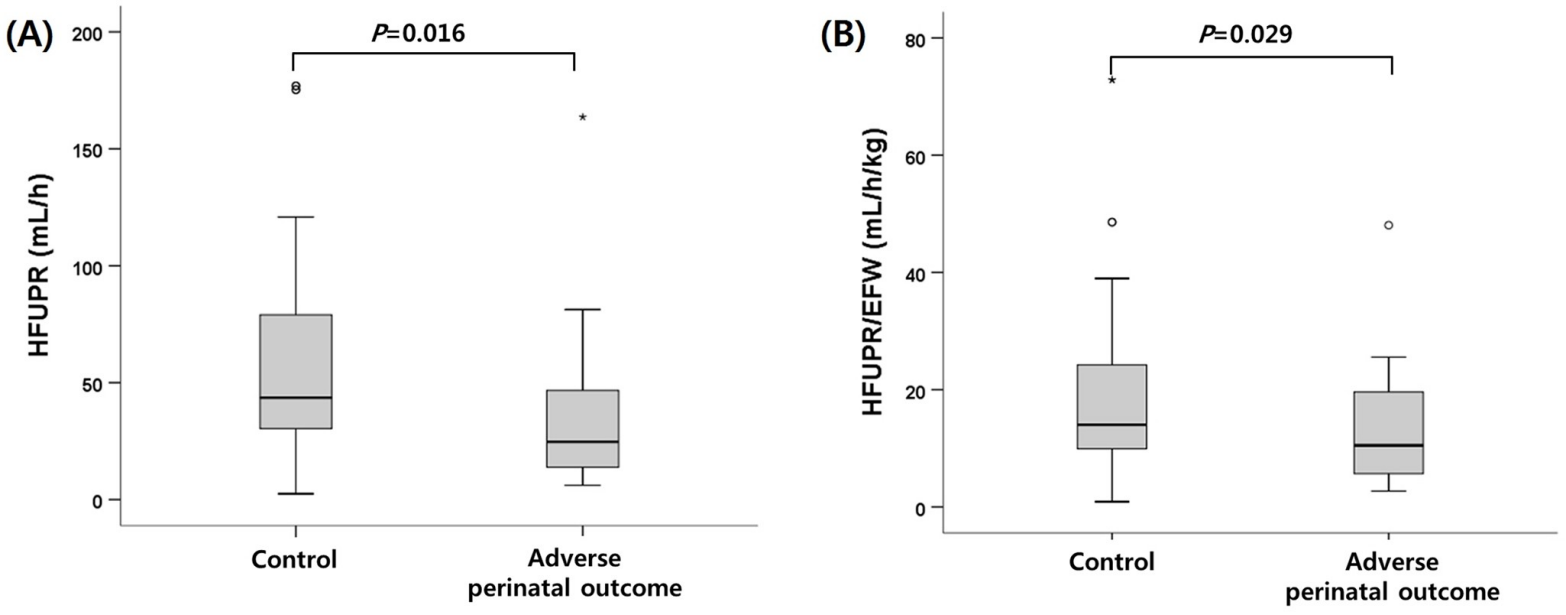
Hourly fetal urine production rate according to adverse perinatal outcomes in all participants. (A) HFUPR in each group. (B) HFUPR/EFW in each group (EFW, estimated fetal weight).

When all participants were divided into two groups according to adverse perinatal outcomes, HFUPR was lower in the adverse perinatal outcome group compared to the control group (median, 24.7 mL/h [IQR 13.4–47.4] vs. 43.6 [29.8–79.0], p = 0.016), and HFUPR/EFW was lower in the adverse perinatal outcome group compared to the control group (median 10.5 mL/h/kg [IQR 5.6–19.8] vs. 14.0 [9.7–24.2], p = 0.029) (Fig 2). However, there was no significant difference of HFUPR in adverse perinatal outcome group compared to the control group among isolated oligohydramnios group (median, 39.5 mL/h [IQR 14.9–47.8] vs. 40.0 [35.8–79.0], p = 0.191). In IUGR group, HFUPR was not found to be significantly different between adverse perinatal outcome group and the control group (median,12.2 mL/h [IQR 8.0–19.8] vs. 24.2 [17.2–31.9], p = 0.062). The intraclass correlation coefficient for intraobserver variability of HFUPR was 0.986 representing good reproducibility.

## Discussion

Fetal urine production is decreased during uteroplacental insufficiency or intrauterine hypoxia. Nicolaides et al. [24] revealed that HFUPR was significantly decreased in preterm IUGR fetuses between 20 weeks’ and 37 weeks’ gestation, and it was correlated with the degree of fetal hypoxia and fetal smallness. Lee et al. [25] quantified HFUPR in uteroplacental insufficiency among hypertensive pregnant women, mostly with normal amniotic fluid levels (43/47) and found that HFUPR was significantly decreased in hypertensive pregnant women with adverse perinatal outcomes compared to those with normal perinatal outcomes, indicating that HFUPR might be a useful predictor of adverse perinatal outcomes in patients with uteroplacental insufficiency. Consistent with previous studies, our study revealed that HFUPR was significantly decreased in the IUGR group, which had more adverse perinatal outcomes than the normal pregnancy and isolated oligohydramnios groups. However, HFUPR was not significantly decreased in the group with isolated oligohydramnios compared to those with normal amniotic fluid levels. Our results suggest that isolated oligohydramnios with normal fetal growth in term pregnancy is not associated with fetal oliguria or fetal hypoxia and support previous reports indicating that routine obstetric interventions may not be needed in isolated oligohydramnios in term pregnancy [10, 11, 26]. Adverse perinatal outcomes were more likely to be related to iatrogenic prematurity due to indicated preterm deliveries in isolated oligohydramnios [27, 28]. Oligohydramnios can occur in normal fetal urination through increased intramembranous absorption with unknown etiology. Recent experimental research revealed the presence of a stimulator for active intramembranous absorption in fetal urine [29]. Therefore, individual fetal risk assessment is needed in women with isolated oligohydramnios at term.

HFUPR has been investigated to assess individual fetal risk in patents with other high-risk pregnancy. Touboul et al. [30] showed that HFUPR was increased in unexplained polyhydramnios and was relatively high in fetuses with an adverse childhood prognosis, although it did not reach statistical significance due to lack of statistical power; this suggests the possibility of HFUPR as a prognostic factor in unexplained polyhydramnios. Maged et al. [21] have been proposed the measurement of fetal urine production as an assessment of fetal well-being in pregnant women with uncontrolled diabetes. In this study, HFUPR was significantly lower in adverse perinatal outcome group compared to those of control among all participants. However, there was no significant difference in HFUPR between adverse perinatal outcome group and control in isolated oligohydramnios and IUGR groups although HFUPR tended to decrease in adverse perinatal outcome group among IUGR. Therefore, future prospective studies are needed to examine the utility of HFUPR for detecting compromised fetus at term.

HFUPR has been assessed by various techniques based on serial measurements of fetal bladder volume using 2D [31, 32] or 3D ultrasonographic measurement [20, 30, 33]. Calculating fetal bladder volume by VOCAL using 3D is easier, more feasible, and superior to 2D ultrasonography for the measurement of fetal bladder volume [34]. HFUPR determined via 3D in normal fetuses at term yielded relatively inconsistent results in this study population. We found that the HFUPR at 37 weeks’ gestation was 59.0 ± 47.3 ml/h, which was consistent with the values of 57.2 ml/h at 36–37 6/7 weeks’ gestation reported by Lee et al. [20] and 54.35 ml/h at 37 weeks’ gestation reported by Peixoto-Filho et al. [33], but lower than 78.4 ml/h at 37 weeks’ gestation reported by Touboul et al. [30]. These variations in HFUPR might be related to fetal biometric differences because our results were most similar to those of Lee et al., which were also conducted on a Korean population [20, 35].

Most commonly used methods to assess amniotic fluid volume are AFI and the single deepest vertical pocket (SDVP) [36]. We used AFI to measure the amniotic fluid in this study, which has been reported to overestimate oligohydramnios over SDVP [37]. Thus, one limitation of our study was that more women may have been diagnosed with oligohydramnios. In fact, ultrasonographic assessment of amniotic fluid using both AFI and SDVP has shown low sensitivity and specificity for diagnosis of oligohydramnios at term pregnancies and reproducibility of both methods was also reported to be low [38–40].

The strength of this study is that, in our knowledge, it is the first report to measure HFUPR using VOCAL in isolated oligohydramnios at term and to compare it with the normal group. Our findings about fetal urine production will help to establish an appropriate treatment for isolated oligohydramnios at term. This study has several limitations. During the process of measuring fetal bladder volumes, the examiner was not blinded and therefore, the bias was not completely excluded although HFUPR was not measured immediately at the time of ultrasound but was calculated later with fetal bladder volumes. This study was limited by small sample size which could make a type II error of a false negative finding. The post hoc analysis showed that statistical power was more than 95% for difference of HFUPR between IUGR groups and other two groups. However, comparison between isolated oligohydramnios and normal group showed a moderate power of 30%. Lastly, limited analysis of detailed intrauterine hypoxic status may exist because fetal Doppler ultrasound was not performed in the isolated oligohydramnios group.

Our study did not find a significant difference in HFUPR between the isolated oligohydramnios group and the normal group, unlike the IUGR group, which showed a decrease in fetal urine production. Our results suggest that there may be a mechanism other than a decrease in fetal urine production in isolated oligohydramnios, and it might not be complicated with uteroplacental insufficiency. However, the difference of HFUPR according to the adverse perinatal outcomes in the isolated oligohydramnios group and the IUGR group was not observed. A further large-scale study is needed to demonstrate with high statistical power that HFUPR is not decreased in isolated oligohydramnios compared to the normal group.

## Conclusion

HFUPR was not significantly decreased in women with isolated oligohydramnios in term pregnancy compared to normal controls. HFUPR was significantly decreased in IUGR groups compared to controls at term pregnancy.

## Supporting information

S1 Dataset (XLSX)

S2 Dataset (XLS)
